# Retraction Note: Mechanism of arterial injury exacerbated by hyperhomocysteinemia in spontaneously hypertensive rats

**DOI:** 10.1038/s41598-024-70879-5

**Published:** 2024-08-27

**Authors:** Lihua Zhang, Rui Xu, Xiaoshan Ma, Xia Zhang, Jun Gong, Zhongliang Li

**Affiliations:** 1 Department of Medicine, Jinan Maternity and Child Care Hospital Affiliated to Shandong First Medical University, Jinan, China; 2 Department of Cardiology, Central Hospital Affiliated to Shandong First Medical University, Jinan, 250013 Shandong People’s Republic of China; 3 Laboratory Department, Jinan Maternity and Child Care Hospital, Jinan, China; 4 Department of Women Healthcare, Jinan Maternity and Child Care Hospital Affiliated to Shandong First Medical University, Jinan, China

Retraction of: *Scientific Reports* 10.1038/s41598-023-28731-9, published online 11 February 2023

The Editors have retracted this Article. An investigation by the journal found that the data used in this study has been previously published^1^ in an article with shared authors. Figure 2, Figure 5, and Figure 6 of the Article display the same data as Figure 2, Figure 4, and Figure 5 of^1^, respectively. Overlap was also noted between images in Figure 3C of the Article and Figure 3 (B1) of the previous publication^1^.

None of the Authors have responded to correspondence from the Editors about this retraction.
